# RETRACTED: Safaee et al. Silencing ZEB2 Induces Apoptosis and Reduces Viability in Glioblastoma Cell Lines. *Molecules* 2021, *26*, 901

**DOI:** 10.3390/molecules31152706

**Published:** 2026-08-04

**Authors:** Sahar Safaee, Masoumeh Fardi, Nima Hemmat, Neda Khosravi, Afshin Derakhshani, Nicola Silvestris, Behzad Baradaran

**Affiliations:** 1 Immunology Research Center, Tabriz University of Medical Sciences, Tabriz 51656-65811, Iran; shar.safaee@gmail.com (S.S.); m.fardi313@gmail.com (M.F.); nima.hemmat1995@gmail.com (N.H.); nedakhosravi74@yahoo.com (N.K.); afshin.derakhshani94@gmail.com (A.D.); 2 Hematology Division, Immunology Department, Tabriz University of Medical Sciences, Tabriz 51656-65811, Iran; 3 IRCCS Istituto Tumori “Giovanni Paolo II” of Bari, 70124 Bari, Italy; 4 Department of Biomedical Sciences and Human Oncology, University of Bari “Aldo Moro”, 70124 Bari, Italy

The journal retracts the article entitled “Silencing ZEB2 induces apoptosis and reduces viability in glioblastoma cell lines” [1], cited above.

Following publication, concerns were brought to the attention of the Editorial Office regarding image integrity and the validity of the findings presented in this article [1].

In accordance with the journal’s standard procedures, the Editorial Office and Editorial Board conducted an investigation that confirmed indications of inappropriate image editing and duplication within Figure 8. Additionally, concerns relating to the validity of a number of citations that had subsequently been retracted could not be sufficiently resolved. While the authors collaborated within this process, raw material meeting the journal’s requirements could not be provided for Editorial Board Evaluation. Consequently, the Editorial Board has lost confidence in the reliability of the findings and has decided to retract this publication [1], as per MDPI’s retraction policy.

This retraction was approved by the Editor-in-Chief of *Molecules*.

The authors have been informed of this retraction and have indicated their disagreement with this decision.
